# Innervation of the cricothyroid muscle by the recurrent laryngeal nerve

**DOI:** 10.1002/hed.24015

**Published:** 2015-07-14

**Authors:** Hiroo Masuoka, Akira Miyauchi, Tomonori Yabuta, Mitsuhiro Fukushima, Akihiro Miya

**Affiliations:** ^1^ Department of Surgery Kuma Hospital Kobe Japan

**Keywords:** recurrent laryngeal nerve (RLN), external branch of the superior laryngeal nerve (SLN), human communicating nerve, cricothyroid muscle, voice impairment

## Abstract

**Background:**

The recurrent laryngeal nerve (RLN) and the external branch of the superior laryngeal nerve (SLN) are generally thought to innervate the endolaryngeal muscles and the cricothyroid muscle (CTM), respectively. Meticulous anatomic studies found communication between these nerves (ie, the human communicating nerve). In this study, we report the innervation of the CTM by the RLN.

**Methods:**

We performed electromyographic studies of 50 patients during thyroidectomy (20 total and 30 hemithyroidectomies). During surgery, the external branch of the SLN, RLN, and vagus nerve were stimulated. Responses were evaluated by visual observation of the CTM and by electromyographies through needle electrodes inserted into the CTM.

**Results:**

Seventy CTMs were evaluated. The RLN stimulation yielded both visible contractions and clear electromyographic responses (>300 µV) in 27 (39%), either response in 24 (34%), and neither response in 19 (27%) of the CTMs. The vagus stimulation gave similar results.

**Conclusion:**

The RLN innervated the CTM at least in 39% cases. © 2015 The Authors. *Head & Neck* Published by Wiley Periodicals, Inc. *Head Neck* **38**: E441–E445, 2016

## INTRODUCTION

One of the major concerns in thyroid surgery is the risk of voice impairment, and, thus, it is important to preserve the nerves and muscles that are fundamental to phonatory function. The laryngeal muscles are innervated by the recurrent laryngeal nerve (RLN) and the external branch of the superior laryngeal nerve (SLN). It is generally thought that the RLN innervates the endolaryngeal muscles and the external branch of the SLN innervates the cricothyroid muscle (CTM).^1^

Meticulous anatomic studies of cadaver larynges revealed terminal branches of the external branch of the SLN reaching the thyroarytenoid muscle and communicating with branches of the RLN in the larynx.^2^, ^3^, ^4^ This branch is called a “human communicating nerve.”^5^ A recent intraoperative nerve monitoring study with an endotracheal tube with surface electrodes revealed the activation of the endolaryngeal muscles in 70% to 80% of the patients when the external branch of the SLN was electrically stimulated.^6^ This phenomenon is used as one of the intraoperative neural monitoring methods for the external branch of the SLN.^6^

During intraoperative neural monitoring for the RLN in thyroid surgery, we have often observed some movements or contractions of the CTMs when the vagus nerve or the RLN was stimulated. We had thought that this phenomenon was secondary to the movement of the larynx caused by the contraction of the endolaryngeal muscles, but we eventually developed the hypothesis that the RLN might reversely innervate the CTM through the human communicating nerve. We conducted electromyographic studies to clarify the cause of the phenomenon, and we report the presence of a reverse communication, innervation of the CTM by the RLN, which may also be related to the phonatory function of the larynx.

## PATIENTS AND METHODS

### Patients

Between June 2013 and December 2013, we performed intraoperative electromyographic studies on consecutive 50 patients who underwent thyroidectomy for thyroid cancer or thyroid nodules suspicious for follicular tumors. We excluded patients with thyroid cancer with massive extrathyroidal extension, clinical node metastasis, or carcinoma >3 cm in size, patients with nodules >4 cm in size, patients with clinical Hashimoto disease or Graves disease, and patients with preoperative vocal cord paralysis. We performed fiber‐optic laryngoscopy preoperatively in all patients to confirm their functioning vocal cords. There were 44 women and 6 men with a mean age of 47.6 years (range, 23–67 years). Twenty of the patients underwent total thyroidectomy and 30 had hemithyroidectomies. During surgery, the external branch of the SLN, RLN, and vagus nerves were electrically stimulated. Responses were evaluated by visual observation of the CTM and by the electromyographic records obtained with a pair of needle electrodes inserted into the CTM and endotracheal tube‐based surface electrodes as well. Thus, 70 sets of the CTM, external branch of the SLN, vagus nerve, and RLN were studied. The present study was approved by the Ethical Committee of Kuma Hospital. Patients gave informed consent for the procedure and for their data to be used.

### Anesthesia

For the surgery, the patient was put under general anesthesia with an endotracheal tube with surface electrodes, and electromyographic records were obtained with the NIM Response 3.0 system (Medtronic, Jacksonville, FL). The tube position was adequately adjusted under flexible fiber‐optic laryngoscopy after the intubation. After the endotracheal intubation with the use of a muscle relaxant, a sufficient dose of sugammadex sodium was injected intravenously to reverse the muscular relaxation, and anesthesia was maintained with sevoflurane and narcotics (remifentanil or fentanyl).

### Surgical techniques

All operations were performed by 1 of the 5 authors. A pair of reference needle electrodes was inserted into the skin around the shoulder. The surgeries were done through a standard collar skin incision. The sternohyoid muscles were dissected at the midline and retracted laterally. The sternothyroid muscles were divided near the insertion to the thyroid cartilage to allow the best exposure of the upper pole of the thyroid lobe. The upper pole of the lobe was retracted laterally and caudally to expand the sternothyroid‐laryngeal triangle.

The sternothyroid‐laryngeal triangle was then searched with a monopolar‐stimulating electrode for a cricothyroid muscular twitch, which is the sign of the presence of the external branch of the SLN.^7^ After the external branch of the SLN was detected, the vagus nerve and the RLN were searched. The vagus nerve was exposed in the carotid sheath. The RLN was detected and exposed in the area caudal to the lower pole of the thyroid lobe. When all 3 of the nerves were exposed, neuromonitoring was performed.

### Neuromonitoring techniques

**Figure 1:**
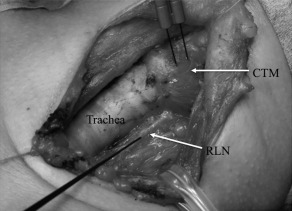
The left thyroid lobe was resected and the left recurrent laryngeal nerve (RLN) was exposed for the explanation of the present study. A pair of electrodes was inserted into the pars rectus of the left cricothyroid muscle (CTM). A monopolar probe stimulated the left RLN. The actual stimulation data for the present study were obtained before resecting the thyroid.

The NIM Response 3.0 system uses an interrupted stimulation technique with 100‐ms impulse duration and 4‐Hz frequency. The nerves were stimulated with a monopolar probe at 1 mA for the external branch of the SLN and the RLN and at 2 mA for the vagus nerve. After the stimulation, the ipsilateral CTM was visually observed for possible movements and electromyographic records were obtained with a pair of needle electrodes directly inserted into the pars recta of the CTM (see Figure 1) and also with endotracheal tube‐based surface electrodes.^8^ We recorded the response amplitudes and the latency of the beginning of the evoked wave from the stimulation. We regarded a clear evoked action potential waveform >300 µV and the latency <10 ms as a positive response.

### Statistical analysis

Statistical evaluations were performed using StatFlex 6.0 software (Artech, Osaka, Japan). For the comparison of continuous variables, Student's *t* test or the Mann–Whitney *U* test was used when appropriate. For the comparison of discrete variables, Fisher's exact test was used. A *p* value < .05 was regarded as significant.

## RESULTS

**Figure 2:**
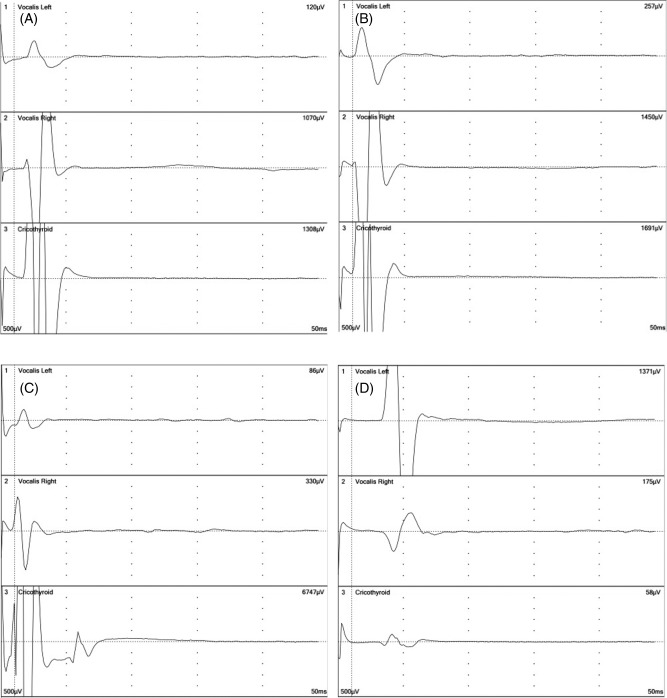
Electromyographic records of the bilateral glottis muscles and the cricothyroid muscle (CTM) in a 44‐year‐old woman. The upper 2 records were obtained through endotracheal tube‐based surface electrodes and the lower record was obtained through a pair of needle electrodes inserted into the muscle. (A) The right vagus nerve was stimulated at 2 mA. (B) The right recurrent laryngeal nerve was stimulated at 1 mA. Note the clear evoked response in the CTM at the same latency for the glottis muscle responses in panels A and B. (C) The right external branch of the superior laryngeal nerve was stimulated at 1 mA, showing an evoked response in the right glottis muscle and a very strong response in the CTM. (D) The left vagus nerve was stimulated at 2 mA. A weak wave from the left CTM was seen at the same latency to the glottis muscle responses.

After the stimulation of the vagus nerve and the RLN, some movement or contraction of the CTM was frequently observed visually, and the electromyographic records through the needle electrodes inserted into the CTM showed various extents of evoked response (Figure 2A, 2B, and 2D). The stimulation of the external branch of the SLN yielded a clear twitch of the CTM, a high amplitude of evoked response through the needle electrodes, and some degree of response of glottis muscles through the surface electrodes based on the endotracheal tube (Figure 2C).

The electromyographic records in Figures 2A to 2D were taken in a 44‐year‐old female patient, showing that her right CTM was clearly innervated by her right vagus nerve and RLN (Figure 2A and 2B), whereas the presence or absence of the innervation on her left side was not clear (Figure 2D).

The time latency between the electrical stimulation and the beginning of the evoked electric wave obtained through the needle electrode inserted into the CTM was quite similar to that for the glottis muscles obtained through endotracheal tube‐based surface electrodes. These data strongly suggested that the response of the CTM was a result of direct innervation and not secondary to the motion of the vocal cord. The latencies after the stimulation of the RLN, right vagus nerve, and left vagus nerve differed because of the difference in the distance from the site of stimulation to the innervated muscle.

After the stimulation of the vagus nerve, some movement or contraction of the CTM was visually observed in 36 of the 70 CTMs (51%; the remaining 34 showed no movement), and evoked amplitude >300 µV was recorded in 46 (66%) CTMs. The remaining 24 showed evoked amplitude <300 µV. Table 1 shows the correlation between the movement of the CTM and the evoked amplitude. Both positive responses were observed in 29 of the 70 CTMs (41%), and both responses were negative in 17 CTMs (24%). Only 1 of the 2 responses was positive in the remaining 24 CTMs. The evoked amplitude from the CTMs with visual movement was significantly larger than that from the CTMs without visual movement (525 ± 236 µV vs 286 ± 205 µV; *p* = .001; Table 2).

**Table 1:** Correlation between the visible movement and the electromyographic response of the cricothyroid muscle when the vagus nerve was stimulated.

	Evoked amplitude		
Movement of CTM	>300 μV	<300 μV	Total no.
Yes	29 (41%)	7 (10%)	36 (51%)
No	17 (24%)	17 (24%)	34 (49%)
Total no.	46 (66%)	24 (34%)	70

Abbreviation: CTM, cricothyroid muscle.

Values are the numbers of CTMs studied. The electromyographic responses were recorded with a pair of needle electrodes inserted into the CTM. The vagus nerve was stimulated at 2 mA. Fisher's exact test: *p* = .0113.

**Table 2:** Visible movement of the cricothyroid muscle and evoked amplitude from the muscle when the vagus nerve was stimulated.

	Evoked amplitude		
Movement of CTM	>300 μV	<300 μV	Total no.
Yes	564 ± 262 μV^*^	201 ± 39 μV	525 ± 236 μV^†^
No	539 ± 170 μV	129 ± 41 μV^*^	286 ± 205 μV^†^

Abbreviation: CTM, cricothyroid muscle.

Values for evoked amplitudes are median ± interquartile range.

The electromyographic responses were recorded with a pair of needle electrodes inserted into the CTM. The vagus nerve was stimulated at 2 mA.

Mann–Whitney *U* test: ^*^ *p* < .001; ^†^ *p* = .001.

The stimulation of the RLN yielded results similar to those of the vagus verve stimulation (Tables 3 and 4). Some movement or contraction of the CTM was visually observed in 36 of the 70 CTMs (51%), and evoked amplitude >300 µV was recorded in 42 CTMs (60%). Both responses were positive in 27 of the 70 CTMs (39%) and both responses were negative in 19 CTMs (27%). The evoked amplitude from the CTMs with visual movement was significantly larger than that from the CTMs without visual movement (433 ± 359 µV vs 272 ± 158 µV; *p* = .01; Table 4).

**Table 3:** Correlation between the visible movement and the electromyographic response of the cricothyroid muscle when the recurrent laryngeal nerve was stimulated.

	Evoked amplitude		
Movement of CTM	>300 μV	<300 μV	Total no.
Yes	27 (39%)	9 (13%)	36 (51%)
No	15 (21%)	19 (27%)	34 (49%)
Total no.	42 (60%)	28 (40%)	70

Abbreviation: CTM, cricothyroid muscle.

Values show the numbers of the CTMs studied. The electromyographic responses were recorded with a pair of needle electrodes inserted into the CTM. The recurrent laryngeal nerve was stimulated at 1 mA. Fisher's exact test: *p* = .0141.

**Table 4:** Visible movement of the cricothyroid muscle and evoked amplitude from the muscle when the recurrent laryngeal nerve was stimulated.

	Evoked amplitude		
Movement of CTM	>300 μV	<300 μV	Total no.
Yes	681 ± 425 μV^*^	229 ± 45 μV	433 ± 359 μV^†^
No	569 ± 328 μV	213 ± 59 μV^*^	272 ± 158 μV^†^

Abbreviation: CTM, cricothyroid muscle.

Values for evoked amplitudes are median ± interquartile range.

The electromyographic responses were recorded with a pair of needle electrodes inserted into the CTM. The recurrent laryngeal nerve was stimulated at 1 mA.

Mann–Whitney *U*‐test: ^*^ *p* < .001; ^†^ *p* = .01.

These results indicated that at least 39% and possibly 76% of the CTMs were innervated by the RLN, probably through reverse communication through the human communicating nerve.

The ratio of the evoked amplitude of the CTM after the vagus nerve stimulation to that obtained after the external branch of the SLN stimulation might suggest the proportion of the innervation of the CTM by the vagus nerve or RLN. The ratio was significantly larger in the CTMs with double‐positive responses compared to that in the CTMs with double‐negative responses (8.5 ± 7.4% vs 1.7 ± 0.9%; *p* = .0001; Table 5). The largest ratio in the present series was 62%. Here, double responses mean visual movement of the CTM and evoked amplitude >300 µV, as described above.

**Table 5:** Proportion of evoked amplitudes stimulated with the vagus nerve compared to those stimulated with the ipsilateral external branch of the superior laryngeal nerve.

Evoked amplitude stimulated with	CTMs with double‐positive responses	CTMs with double‐negative responses	Significance
A: Vagus (μV)	647 ± 296	129 ± 44	*p* < .0001
B: External branch of the SLN (μV)	7237 ± 3723	7977 ± 3601	*p* = .763
A to B ratio (%)	8.5 ± 7.4	1.7 ± 0.9	*p* = .0001

Abbreviations: CTM, cricothyroid muscle; SLN, superior laryngeal nerve.

Values for evoked amplitudes are median ± interquartile range.

Electromyographic responses were recorded with a pair of needle electrodes inserted into the CTM. The external branch of the SLN was stimulated at 1 mA and the vagus at 2 mA.

## DISCUSSION

In the present study, about 40% of the CTMs showed visual movement or contraction and a clear electromyographic response after the stimulation of the vagus nerve or the RLN. Another 34% of the CTMs showed either visual movement or an electromyographic response. The discrepancies in the responses in these cases might be caused by innervation of only a small portion of the CTM and inappropriate location of the needle electrodes, or partial innervation that is sufficient to yield an electromyographic response but insufficient to cause macroscopic contraction of the muscle. Thus, the present results revealed that the RLN innervated the CTM in at least approximately 40% of the cases and that this may be true in an additional 34% cases. The human communicating nerve between the external branch of the SLN and the RLN can conduct neural stimulation not only from the external branch of the SLN to the glottis muscles but also from the RLN to the CTM.

Martin–Oviedo et al^9^ stimulated the RLN during total laryngectomy in 13 patients and observed an evoked response from the CTM in 7 of the patients. However, they did not report on the relationship between the evoked response and the visual contraction of the CTM. In addition, they did not stimulate the vagus nerve. Therefore, the present study is the first report that revealed the innervation of the CTM by the RLN and vagus nerve in a large patient series.

The physiological implications of the phenomenon of innervation of the CTM by the RLN are not clear. Individuals with this “communicating” innervation might have milder symptoms of paralysis of the external branch of the SLN if the external branch of the SLN is injured, because part of the CTM would remain innervated by the RLN. In their anatomic necropsy studies, Maranillo et al^3^ described the presence of small branches communicating between the external branch of the SLN and the RLN, and they suggested that the branches might act as a preventive for atrophy of the CTM in cases of external branch of the SLN paralysis because the “communicating” innervation might work as an alternative route from the RLN to the CTM.

On the other hand, individuals with this innervation might have greater symptoms in their voice impairment if the RLN is injured, because part of the CTM would also become paralytic or weakened. In the present study, the median ratio of the evoked amplitude of the CTM after the stimulation of the RLN to that obtained after the stimulation of the external branch of the SLN was 8.5%, and in the largest case it was 62%. This communicating innervation could thus have significant clinical implications.

Patients with RLN paralysis have variable degrees of voice impairment.^10^ Our patients with unilateral vocal cord paralysis had maximum phonation times that were significantly shorter than those of the normal subjects and ranged rather widely from 5 to 15 seconds in male patients and from 3 to 12 seconds in female patients.^11^ Paralytic vocal cords are usually seen at a paramedian position, but they may be found in different positions, for which no clear explanation has been made.^10^ There are reports arguing whether the status of the CTM influences the vocal cord position in the RLN paralysis.^12^, ^13^ Thus, these phenomena might be related to the presence and the extent of the inverse innervation of the CTM by the RLN. Further studies are needed to classify the possible effect on phonatory function of the inverse innervation reported in the present article.

## CONCLUSION

The RLN innervated at least approximately 40% of the CTMs and possibly an additional 34% of the 70 CTMs examined. Contraction of the CTM by the electrical stimulation of the vagus nerve and the RLN was visibly observed and confirmed by electromyography. The human communicating nerve between the external branch of the SLN and the RLN can conduct neural stimulation not only from the external branch of the SLN to the endolaryngeal muscles but also from the RLN to the CTM.
